# The Link between Parenting Behaviors and Emerging Adults’ Relationship Outcomes: The Mediating Role of Relational Entitlement

**DOI:** 10.3390/ijerph19020828

**Published:** 2022-01-12

**Authors:** Octav-Sorin Candel

**Affiliations:** Faculty of Psychology and Educational Sciences, Alexandru Ioan Cuza University of Iasi, 700554 Iasi, Romania; octav.candel@uaic.ro; Tel.: +40-0232-20-1301

**Keywords:** parenting, romantic relationships, entitlement

## Abstract

Previous research shows a link between parenting and children’s characteristics and interpersonal behaviors. However, little is known about the ways in which parenting tactics affect children’s romantic relationships and whether the children’s characteristics can mediate these associations. With this study, the aim was to test the associations between parents’ helicopter parenting/autonomy-supportive behaviors and emergent adults’ relational satisfaction and couple conflict. In addition, it was tested whether the sense of relational entitlement (excessive and restricted) mediated the links. Two hundred and twelve emergent adult–parent dyads participated in this study. Mediation analyses showed that parental autonomy-supportive behaviors had indirect effects on both the relational satisfaction and the couple conflict reported by the emerging adults through excessive relational entitlement. The link was positive for the former couple-related outcome and negative for the latter one. Helicopter parenting was not related to any variable reported by the emerging adults. In conclusion, positive parenting can increase relational stability and well-being by diminishing some potentially negative psychological characteristics of emerging adults.

## 1. Introduction

Parents, whose one important responsibility is raising children, use different parental practices [1]. Although parental socialization is over when the adolescent child reaches the adult age, the impact of these previous family experiences could be crucial in adult life [2,3]. Parental practices are strongly related to the individuals’ development and well-being throughout their lives. While a positive parenting style leads to various indicators of well-adjustment during emerging adulthood, overinvolved or highly controlling parenting has the opposite effect, leading to a number of less fortunate outcomes [4,5,6]. Helicopter parenting (parents’ overinvolvement in the lives of their children, including such behaviors as solving problems and crises for the children) was associated with lower psychological well-being and satisfaction with life [5,7,8]. On the contrary, parental autonomy support was related to better psychological well-being [6,9,10,11]. One particular point of interest for young adults and an important indicator of adjustment is the formation of romantic relationships. According to the development of early adult romantic relationships model, early family relationships are crucial for the later functioning of young adults’ romantic relationships [12]. Confirming this assumption, previous studies showed that the interactions with their parents were related to both positive and negative aspects of the emerging adults’ romantic relationships [13,14]. The first goal of this study is to assess the links between the parents’ perceptions of their parenting practices and their children’s relational satisfaction and couple satisfaction. A second goal of the study is to test a possible mediator of the proposed relationships. Previous studies show that psychological entitlement is related to both parenting and relational outcomes [15,16,17]; thus, this study introduces the form of entitlement most specific to the romantic domain, the sense of relational entitlement [18].

### 1.1. Parenting Practices and Romantic Relationships

Parental practices are associated with a number of outcomes regarding the children’s success and experiences in their romantic relationships. For example, higher use of helicopter parenting on the part of the parents was related to a reduced desire of being involved in a romantic relationship for their children. While the young adults who experienced more helicopter parenting still considered marriage as being important, they also expressed their wish to remain single for a longer period of time and viewed being single as more advantageous than being married [19]. Moreover, the level of parental psychological control was negatively related to interpersonal competence in romantic relationships and with reduced empathy in emerging adults [20,21]. 

Regarding the positive predictors of the quality of romantic relationships, one study found that positive parenting strategies (parental involvement, acceptance, appropriate strictness) were associated with more mutuality and more relational satisfaction in adolescent relationships [22]. In addition, effective parenting was associated with better relationship problem-solving skills [14]. More specifically for this study, autonomy-supportive parenting predicted more adaptive emotion regulation and intimacy [23].

The associations between parental practices and the conflict experienced by young adults in their romantic relationships also received some attention in previous studies. Effective parenting was shown to help children to find better solutions for their problems, and this ability put them at lower risk for relationship violence [14]. Parental overinvolvement in the child’s dating life (which can be seen as a form of helicopter parenting) was related to later reports of experiencing intimate partner violence during adulthood [24]. Finally, while harsh parenting was positively related to aggressiveness towards the romantic partner, supportive parenting had an inverse relationship with hostile behaviors in romantic relationships [25]. 

Most of these studies are, however, biased due to the inclusion of only one source of data, namely, the child or young adult. In this study, the parents’ perception of their parenting style was measured, while all the other variables were measured using the emerging adults’ reports. By doing so, the intension was to expand the current view by including some variables measured at the level of the family of origin. This could be important because potential parenting interventions could target the parents’ behaviors and thus should be based on the parents’ view of such behaviors. 

The first two hypotheses were based on the theoretical perspective proposed by the development of early adult romantic relationships model [12], which states that the relational outcomes of young adults can be related to pre-relational circumstances and interactions, including relationship-promotive or -inhibiting experiences in the family of origin during childhood and adolescence. Among these, the parenting style can be associated with various attributes of the emerging adult’s romantic relationships and relationship success. Starting from this model and from the aforementioned empirical findings, the first two hypotheses were proposed:

**Hypothesis** **1.** *The higher use of helicopter parenting practices as assessed by the parents will be associated with lower levels of relational satisfaction and higher levels of relational conflict for emerging adults.*

**Hypothesis** **2.** *The higher use of autonomy-supportive behaviors as assessed by the parents will be associated with higher levels of relational satisfaction and lower levels of relational conflict for emerging adults.*

### 1.2. The Mediating Role of Relational Entitlement

The development of early adult romantic relationships model [12] proposes that the relationship-promoting and -inhibiting experiences in the family of origin (for example, the parenting style) can influence the individual characteristics of the young adult. These can act as particular competencies that can promote an individual’s success in a romantic relationship. Among them, one can find entitlement. Psychological entitlement (the belief that one deserves more than others as well as the expectations for special treatment) has been seen, for a long time, as a facet of narcissism [26]. However, more recently, narcissism and entitlement have been conceptualized as different, although related, constructs [27]. In this context, the concept of sense of relational entitlement was developed, referring strictly to those expressions of entitlement appearing in the romantic domain, and it can be defined as the extent to which a person expects that their needs and wishes will be fulfilled by the romantic partner, and as a person’s affective and cognitive responses to a romantic partner’s failure to fulfill these needs and hopes [18]. Similar to the broader concept of psychological entitlement, relational entitlement can also have two expressions: one excessive (the individual believes that their needs must be fulfilled regardless of the needs or emotional state of their partner) and one restricted (the individual is unassertive, timid, and less self-assured in demanding to have their needs fulfilled) [18,28].

The link between parenting and children’s psychological entitlement is mostly clear throughout the literature. Overinvolved parenting was associated with more psychological entitlement for the child [15,16]. Moreover, inconsistent discipline and poor monitoring were positively related to child’s entitlement rage [29]. On a more specific domain, both parental psychological control and permissive parenting were associated with greater academic entitlement in children and young adults [30,31]. Although relational entitlement was not specifically targeted by any of these studies, one can consider that the previous literature provides sufficient evidence to support the expectations regarding a possible link between helicopter parenting/autonomy-supportive behaviors and emerging adults’ relational entitlement.

On the contrary, the associations between relational entitlement and relational satisfaction were established by more studies. Cross-sectional, longitudinal, and experimental studies showed that both forms of relational entitlement (excessive and restricted) have significant and negative associations with the quality of one’s relationship, with the link between excessive entitlement and satisfaction being stronger [17,32,33]. Finally, more excessive psychological entitlement is related to higher levels of conflict in interpersonal and romantic relationships [34,35,36,37].

By taking these established relationships into account, the final two hypotheses were as follows:

**Hypothesis** **3.** *Emerging adults’ relational entitlement (excessive and restricted) will mediate the link between helicopter parenting as assessed by the parents and emerging adults’ relational outcomes (relational satisfaction and relational conflict).*

**Hypothesis** **4.** *Emerging adults’ relational entitlement (excessive and restricted) will mediate the link between autonomy-supportive behaviors as assessed by the parents and emerging adults’ relational outcomes (relational satisfaction and relational conflict).*

## 2. Materials and Methods

### 2.1. Participants

Two hundred and twelve emerging adult–parent dyads participated in this study. Among the emerging adults, 20 were men and 192 were women. Their mean age was 21.23 years old (SD = 1.77, min. = 19, max. = 30). One hundred and ninety-nine participants identified themselves as heterosexual (93.9%), two as homosexuals (0.9%), and 11 as bisexuals (5.2%). In this sample, 81 (38.2%) were not in a romantic relationship at the time of the study, while 131 (61.8%) were involved in a romantic relationship at the time of the study. Among those who were single, the mean length of their last relationship was 1.41 years (SD = 1.63 years). Among those who were in a relationship, its mean length was 2.42 years (SD = 1.83 years). The participants who were not in a relationship at the time of the study answered the measures about their last romantic relationship. Two hundred and three participants (95.8%) had finished high school, six (2.8%) had a bachelor’s degree, and 3 (1.4%) had a master’s degree. In terms of religion, 177 participants were Orthodox Christians (83.5%), 19 were Catholics (9%), four were Protestants (1.9%), and 12 were atheists/agnostics (5.7%).

Among the parents, eight participants were men and 204 were women. Their mean age was 41.17 years old (SD = 5.01, min. = 37, max. = 69). In this sample, 16 parents were not married and 196 were married at the time of the data collection. For those who were married, the mean length of marriage was 23.38 years (SD = 8.25 years). Most parents had a high school degree (N = 126; 59.4%), 27 had finished middle school (12.7%), 45 had a bachelor’s degree (21.2%), 13 a master’s degree (6.1%), and one had a doctoral degree (0.5%). In terms of religion, 188 parents were Orthodox Christians (88.7%), 20 were Catholics (9.4%), and four were Protestants (1.9%).

### 2.2. Procedure

The research was approved by the Research Ethics Committee at Alexandru Ioan Cuza University. The questionnaires were distributed to students enrolled in two undergraduate programs and to their parents. While the study mostly targeted the mothers, the participation of the fathers was also allowed when the mother was not available to participate. Participation was voluntary for both the students and their parents. The students received academic credits for their involvement. The parents were not rewarded.

### 2.3. Measures

The Sense of Relational Entitlement Scale (SRE). The Romanian version of the SRE was used to measure the children’s relational entitlement [38]. The scale contains 18 items, rated from 1 (not at all) to 7 (very much). The scale offers scores for assertive entitlement (seven items), excessive entitlement (eight items), and restricted entitlement (three items). However, due to the lack of evidence regarding the link between assertive entitlement and relational satisfaction [28], only the excessive (e.g., “When my partner frustrates me, I contemplate ending the relationship”) and the restricted (e.g., “Sometimes I feel I am not good enough for my partner”) dimensions were used. Both scales showed adequate internal consistency. For excessive relational entitlement, α = 0.85; for restricted relational entitlement, α = 0.78.

Couple Satisfaction Index 4 (CSI-4). The CSI-4 was used to measure the children’s relational satisfaction [39]. The scale contains 4 items (e.g., “Please indicate the degree of happiness, all things considered, of your relationship”). The CSI-4 was created by selecting the best items from the already existing measures of satisfaction. Respondents indicated how content they feel/felt in their current or past romantic relationship on a 7-point Likert scale for one item and a 6-point Likert scale for the others. For this scale, the α =.94, showing very good internal consistency.

Conflict scale. The conflict from the emerging adults’ romantic relationship was measured with a scale containing six items (e.g., “I feel like all my partner and I do is fight”) rated from 1 (strongly disagree) to 7 (strongly agree) [40]. The scale showed very good internal consistency (α = 0.89).

Helicopter Parenting and Autonomy-Supportive Behaviors Scale [41]. This scale was used to measure the parents’ parenting style and autonomy-supportive behaviors. It contains 15 items, rated from 1 (strongly disagree) to 6 (strongly agree). The scale was initially developed to measure the children’s perception of their parents’ behaviors with items formulated using the third person (e.g., “My mother had a say in what major I chose”). However, the items were transformed to reflect the parent’s perception of their own behaviors during their children’s childhood and adolescence; thus, for this study, they were formulated using first person (e.g., “I had a say when my child chose his/her major”). The procedure is similar to that of previous studies with adult children [2]. The items are similar to those for children and adolescents, but the statements are in the past tense [3]. The scale measures two dimension, namely, helicopter parenting (9 items, e.g., “I regularly wanted my child to call or text me to let me know where he/she was”) and autonomy-supportive behaviors (5 items, e.g., “I encouraged my child to discuss any academic problems he/she was having with a professor”). The latter dimension originally contained six items. One item (“I encouraged my child to choose his/her own classes”) was removed because it was not suitable for Romanian parents, given that in Romania all the classes are mandatory. The helicopter parenting subscale showed an adequate internal consistency (α = 0.74). The autonomy-supportive behaviors showed a lower level of internal consistency (α = 0.65). However, the mean inter-item correlation was 0.29, which was adequate, being between the limits of 0.15 and 0.50.

Demographic data for both emerging adults (gender, age, relational status, present or prior relationship length, education, religion, and sexual orientation) and parents (gender, age, relational status, relationship length, education, and religion) were reported. All the items are presented in Appendix A.

### 2.4. Statistical Analyses

First, the descriptive statistics and the correlational analysis were performed using SPSS 21. Second, a series of mediation analyses was computed to verify whether the emerging adults’ relational entitlement mediated the relationship between the parents’ perceived parenting style and the emerging adults’ relational outcomes. Model 4 from Process, an SPSS macro, was used for this analysis. Bootstrapping with 5000 re-samples was used to obtain parameter estimates of the specific indirect effects. The 95% confidence intervals (CIs) were used to determine whether these effects were statistically significant: if the 95% CI does not contain zero, then the indirect effect is considered statistically significant and mediation has been demonstrated.

## 3. Results

### 3.1. Preliminary Results

The means and standard deviations for each variable are presented in Table 1. The normality of the distribution was assessed by using skewness and kurtosis. Given that the values for both indexes were between −2 and 2 for all the variables, their distributions were normal [42].

### 3.2. Correlation Analyses

Pearson product correlations were used to verify the associations between the variables (see Table 2). Among the parents’ variables, a significant and positive association was found between their helicopter parenting and their autonomy-supportive behaviors. When the parents showed higher levels of helicopter parenting, they also showed more autonomy-supportive behaviors. This correlation showed a low effect size, according to Cohen’s criteria [43]. Among the emerging adults’ variables, excessive relational entitlement showed significant positive correlations with conflict and negative correlations with relational satisfaction. The effect sizes were large. Finally, a significant and negative correlation was found between the parents’ autonomy-supportive behaviors and the emerging adults’ level of excessive relational entitlement. The effect size was low.

### 3.3. Mediation Analysis

The first intention was to compute a series of mediation models with either parents’ helicopter parenting or parents’ autonomy-supportive behaviors serving as predictors, emerging adults’ excessive and restricted relational entitlement as mediators, and emerging adults’ relational satisfaction and conflict as outcomes. However, due to the lack of significant correlations between parents’ helicopter parenting and the potential mediators or outcomes, only the parents’ autonomy-supportive behaviors were used as a potential predictor. As such, Hypotheses 1 and 3 were rejected. Moreover, this variable was not significantly associated with restricted entitlement. Thus, two mediation models were analyzed. In each model, the emerging adults’ relational status (single vs. in a relationships) was included a control variable.

Hypotheses 2 and 4 were tested using the following two models. The first model contained the parents’ autonomy-supportive behaviors as a predictor, the emerging adults’ excessive entitlement as a mediator, and the emerging adults’ relational satisfaction as the outcome. Parents’ autonomy-supportive behaviors were significantly and negatively associated with emerging adults’ excessive entitlement (β = −0.13; *p* = 0.04). Further, emerging adults’ excessive entitlement was significantly and negatively associated with their couple satisfaction (β = –0.55; *p* < 0.001). Emerging adults’ relational status was significantly associated with their reported relational satisfaction (β = 0.42; *p* < 0.001), but not with their level of excessive relational entitlement (β = −0.08; *p* = 0.20) Although the direct effect of parents’ autonomy-supportive behaviors on emerging adults’ relational satisfaction was not significant (β = 0.02; *p* = 0.60), the indirect effect through the emerging adults’ excessive entitlement was significant (β = 0.07; CI (0.01, 0.14)).

The second model contained the parents’ autonomy-supportive behaviors as a predictor, the emerging adults’ excessive entitlement as a mediator, and the emerging adults’ couple conflict as the outcome. Emerging adults’ excessive entitlement was significantly and positively related to their couple conflict (β = 0.65; *p* < 0.001). Emerging adults’ relational status was significantly associated with their reported couple conflict (β = −0.17; *p* < 0.01). The direct effect of parents’ autonomy-supportive behaviors on emerging adults’ couple conflict was not significant (β = −0.01; *p* = 0.86), the indirect effect though the emerging adults’ excessive entitlement was significant (β = −0.09; CI (−0.17, −0.01)). Given that the results showed only a significant indirect relationship between parental autonomy-supportive behaviors and emerging adults’ relational satisfaction and couple conflict, Hypothesis 2 was partially confirmed. Hypothesis 4 was also partially confirmed due to the emerging adults’ excessive relational entitlement role as a mediator of the aforementioned relationships. Their restricted relational entitlement, however, was not a significant mediator.

## 4. Discussion

This study had two main objectives. Firstly, to determine whether negative (helicopter parenting) and positive (autonomy-supportive behaviors) parenting practices as assessed by the parents are linked to some relational outcomes (relational satisfaction and couple conflict) experienced by emerging adults. Secondly, to test the mediating role of emerging adults’ relational entitlement in the aforementioned relationships.

Helicopter parenting was not significantly related with the emerging adults’ relational satisfaction or couple conflict. These findings determined the rejection of the first hypothesis. While most previous studies found negative consequences for helicopter parenting [6,7,8,20,21], few, if any, were interested in the same outcomes as in this paper. One study found that the young adults who experienced helicopter parenting were more inclined to postpone finding a romantic partner and marrying [19]. However, in the same study, the participants viewed marriage as important as those who had not experienced helicopter parenting did. Thus, overinvolved parenting seems to affect some views and behavior regarding romantic relationships, but not all of them. Among the latter, relational satisfaction and couple conflict were two outcomes seemingly independent of helicopter parenting. Another possible explanation for the current findings is that helicopter parenting is rather contextual and infrequent [44]. Moreover, some behaviors specific to helicopter parenting might be practiced by the parents who, otherwise, use many positive parenting tactics [44]. Even in this study, helicopter parenting had a significant and positive correlation with autonomy-supportive behaviors, similar to the one found by Schiffrin and colleagues [41]. Theoretically, helicopter parenting could be considered as the opposite of parental support [45]. Parents who tend to use practices related to helicopter parenting (e.g., psychological control) tend not to foster autonomy granting [46]. Nevertheless, at least for the measures used in the present study to capture parenting, a positive relationship between helicopter parenting and parental autonomy support was found, confirming the same result offered by the authors of the questionnaire in their original paper [41]. It is possible that the measure of helicopter parenting could be, in fact, about parental control [45]. Additionally, a recent study found that 77% of mothers and 50% of fathers had a “warm helicopter parenting” style, as opposed to the “controlling helicopter parenting” style reported by less than 20% of the parents [47]. Warmth is an important moderator of the link between helicopter parenting and the children’s negative outcomes, most of them appearing in the absence of warm parenting [5]. In conclusion, not all helicopter parenting is bad, and most parents are combining it with positive behaviors. Given that many parents do so, it is probable that the parents from the current sample also used “warm helicopter parenting”, thus buffering the more negative effects of their controlling behaviors.

The second hypothesis, regarding the link between autonomy-supportive behaviors and relational outcomes, was partially supported. Indeed, there were significant effects of parental autonomy-supportive behaviors on both relational satisfaction and couple conflict, but these were only indirect. In both cases, the mediator was the emerging adults’ excessive relational entitlement. Thus, the fourth hypothesis was partially confirmed. The self-determination theory [48] can be used to explain the link between parental autonomy-supportive behaviors and excessive entitlement. Parental autonomy-supportive behaviors lead to higher satisfaction of the basic psychological needs [6]. When their autonomy, competence, and relatedness need is satisfied on a regular basis, children might develop an appropriate and more adaptive sense of relational entitlement. Thus, they consider that their close ones are able to fulfill their needs without feeling an exaggerated impulse to ask for it. In addition, when parents offer their children the appropriate level of autonomy, they also lower the chances for the child to develop an inflated sense of deservingness. By acting like their children are (at least partially) responsible for their behavior and supporting their autonomy, parents help their children to become self-efficient in solving some of their problems rather than believing they deserve special treatment from those around them [16,49]. While the relationship between parental autonomy-supportive behaviors and excessive relational entitlement was firstly established by this study, the results also confirmed previous findings linking excessive entitlement, lower relational satisfaction, and higher couple conflict [17,33]. One study found that entitlement inflates self-image goals and that this selfish agenda leads to potential interpersonal conflict [35]. The same might be true in romantic relationships, where one partner’s need to center all the couple’s attention on themselves can lead to conflict as well as to lower satisfaction when this need is not fulfilled.

Previous studies showed that helicopter parenting might be detrimental to young adults’ psychological and relational development [50]. This study, however, does not support these findings. Still, the results show that parental use of autonomy-supportive behaviors is related to an increase in relational satisfaction and a decrease in couple conflict for their children. Although indirect, this association underlines the positive effects of offering sufficient autonomy to children. Using a parenting style characterized by both high autonomy and high monitoring seems to be most beneficial for children, as they experience the highest levels of life satisfaction and positive affect [51]. By taking into account the results, high autonomy is also related to higher levels of success in romantic relationships. This paper also confirms some parts of the development of early adult romantic relationships model [12], by providing some evidence for the link between parenting style, as it is assessed by the parent (seen as a characteristic of the family of origin), and emerging adults’ relational entitlement, a specific interpersonal skill or competency predicted to influence the probability of success in early adult romantic relationships. When children develop in a warm and supportive environment, with sufficient nurturant parenting, they also develop suitable competencies that help them succeed in their romantic relationships. One such competency can be an ideal relational entitlement style, one that is characterized by realistic expectancies of need fulfillment, optimal assertiveness, and care towards the needs of the partner. Consequently, such skills provide an increase in relational success, namely, more satisfaction and less conflict. Still, longitudinal studies are needed to establish the link between other positive parenting practices and relational quality. Emerging adults’ romantic relationships are known to be rather unstable, with many young adults being involved in on-and-off or serial relationships [52]. Still, relationship satisfaction is characterized by a large degree of interindividual variability, with many personal characteristics shaping the trajectory of a couple’s satisfaction [53,54]. Relational entitlement seems to be among them. When emerging adults experience an increased level of excessive entitlement, the trajectory of their relational outcomes (satisfaction and conflict) seems to follow the decline that can be expected from their age group. However, an appropriate sense of entitlement can lead to an increase in positive relational outcomes.

One of the main implications of this study is that it shows that good parenting promotes romantic competency and success through the development of particular romantic competencies. Moreover, this was found using the parents’ assessments of their parenting style. Because the way in which parents see their parenting seems to be important, using programs promoting optimal parenting, which includes the use of autonomy-supportive behaviors, might have additional benefits beyond those already discussed by the literature [55].

Although this study enhances the literature, several limitations must be noted. Firstly, only self-reported questionnaires that assessed the parents’ perception of their parenting style were used. Some studies show that parents and children might disagree when assessing parenting [56,57]. In addition, observation studies regarding parenting practices also lead to different results [58]. Thus, it would be important for future studies to use a more comprehensive method of measuring parenting. Secondly, since mothers’ and fathers’ parenting styles tend to differ, the fact that most of the parent sample was composed of mothers can also act as a limitation [47]. Testing both parents can offer a different view on the family system and on how the relationships between parents and children act as determinants of children’s relational outcomes. Thirdly, based on the cross-sectional results, a clear causal relationship between the variables cannot be established. Finally, this study took into account only Romanian parent–child dyads. Moreover, the children were students. Other studies showed that the use of different parenting styles tends to vary in different cultures [59]. Cross-cultural studies or studies using various populations are needed to show that these findings are significant in other cultures or other populations.

## 5. Conclusions

This study had two aims: to test the associations between parenting practices and the emerging adults’ relational outcomes and to verify whether the emerging adults’ relational entitlement mediates those associations. The results showed an indirect association between parental autonomy-supporting behaviors and relational satisfaction, as well as between parental autonomy-supporting behaviors and couple conflict. Both relationships were mediated by excessive relational entitlement. Thus, the study shows the importance of using positive parenting tactics for the adults’ psychological and interpersonal development.

## Figures and Tables

**Table 1:** Summary statistics for the variables included in the study.

	M	SD	Skewness	Kurtosis
P Helicopter Parenting	33.18	8.07	−0.09	−0.21
P Autonomy-Supportive Behaviors	25.10	3.85	−1.00	1.24
C Excessive Entitlement	17.50	6.50	0.94	0.53
C Restricted Entitlement	7.90	3.07	0.20	−0.80
C Relational Satisfaction	19.77	4.56	−0.95	0.14
C Couple Conflict	16.11	7.90	0.96	0.21

Note: P = parents’ variables; C = children’s variables.

**Table 2:** Correlation analysis.

	1	2	3	4	5
1. P Helicopter Parenting					
2. P Autonomy-Supportive Behaviors	0.29 ***				
3. E Excessive Entitlement	−0.06	−0.14 *			
4. E Restricted Entitlement	−0.05	−0.03	0.29 ***		
5. E Relational Satisfaction	0.09	0.10	−0.59 ***	−0.07	
6. E Couple Conflict	−0.08	−0.10	0.66 ***	0.10	−0.71 ***

Note: P = parents’ variables; E = emerging adults’ variables; *** *p* < 0.001; * *p* < 0.05.

## Data Availability

The data presented in this study are available on request from the corresponding author.

## Appendix A

### Appendix A.1. The Sense of Relational Entitlement Scale (SRE)

1. I’m often preoccupied with the question of whether my partner is good enough for me.
2. Sometimes I feel my partner is not good enough for me.
3. I am obsessed with my partner’s faults.
4. When my partner frustrates me, I contemplate ending the relationship.
5. When my partner frustrates me, I start thinking about new relationships.
6. When my partner hurts me, I’m immediately filled with a sense of distrust.
7. I often feel I deserve to get more than I do in my relationship.
8. In my relationship, I’m sometimes filled with a kind of rage that I hardly ever experience in daily life.
9. Sometimes I feel I am not good enough for my partner.
10. I’m often preoccupied with the question of whether I deserve my partner.
11. I feel my partner deserves to get more than he or she does in our relationship.

Items 1 to 8 are used to compose the excessive relational entitlement score. Items from 9 to 11 are used to compose the restricted entitlement score.

### Appendix A.2. Couple Satisfaction Index 4

1. Please indicate the degree of happiness, all things considered, of your relationship (0—extremely unhappy; 6—perfect)
2. I have a warm and comfortable relationship with my partner (1—not at all true; 6—completely true)
3. How rewarding is your relationship with your partner? (1—not at all; 6—completely)
4. In general, how satisfied are you with your relationship? (1—not at all; 6—completely)

### Appendix A.3. Conflict Scale

1. My partner and I have a lot of disagreements.
2. I feel like all my partner and I do is fight.
3. There is a lot of conflict in my relationship.
4. I am often irritated by my partner.
5. My partner and I are always in agreement on major issues.
6. It is rare that my partner and I get in a big argument.

### Appendix A.4. Helicopter Parenting and Autonomy Supportive Behaviors Scale

1. I had a say when my child chose his/her major.
2. I encouraged my child to discuss any academic problems he/she was having with a professor.
3. I monitored my child’s exercise schedule.
4. When he/she was home with me, my child had a curfew (a certain time that he/she must be home by every night)
5. I gave my child tips on how to shop for groceries economically.
6. I encouraged my child to make his/her own decisions and take the responsibility for the choices he/she has made.
7. I regularly wanted my child to call or text me to let me know where he/she was.
8. I encouraged my child to deal with any interpersonal problems between himself/herself and his/her roommate or friends on his/her own.
9. If my child was to receive a low grade that he/she felt was unfair, I would have called the professor.
10. I monitored my child’s diet.
11. I monitored with whom my child spent time.
12. I encouraged my child to keep a budget and manage his/her own finances.
13. I called my child to track his/her schoolwork (i.e., how he/she was doing in school, what his/her grades were like, etc.).
14. If my child has an issue with his/her roommate, I would try to intervene.

Items 1, 3, 4, 7, 9, 10, 11, 13 and 14 are used to compose the helicopter parenting score. Items from 2, 5, 6, 8 and 12 are used to compose the autonomy supportive behaviors scale.
